# Structural Basis of a Human Neutralizing Antibody Specific to the SARS-CoV-2 Spike Protein Receptor-Binding Domain

**DOI:** 10.1128/Spectrum.01352-21

**Published:** 2021-10-13

**Authors:** Mei Yang, Jiaqi Li, Zhaoxia Huang, Heng Li, Yueming Wang, Xiaoli Wang, Sisi Kang, Xing Huang, Changwen Wu, Tong Liu, Zhenxing Jia, Junlang Liang, Xiaohui Yuan, Suhua He, Xiaoxue Chen, Zhechong Zhou, Qiuyue Chen, Siqi Liu, Jing Li, Huiwen Zheng, Xi Liu, Kenan Li, Xiaojun Yao, Bin Lang, Longding Liu, Hua-Xin Liao, Shoudeng Chen

**Affiliations:** a Molecular Imaging Center, Guangdong Provincial Key Laboratory of Biomedical Imaging, The Fifth Affiliated Hospital, Sun Yat-sen University, Zhuhai, China; b Zhuhai Trinomab Biotechnology Co., Ltd., Zhuhai, China; c Institute of Medical Biology, Chinese Academy of Medical Sciences & Peking Union Medical College, Kunming, China; d Institute of Biomedicine, Jinan University, Guangzhou, China; e Department of Infectious Diseases, The Fifth Affiliated Hospital, Sun Yat-sen University, Zhuhai, China; f Dr. Neher’s Biophysics Laboratory for Innovative Drug Discovery, State Key Laboratory of Quality Research in Chinese Medicine, Macau Institute for Applied Research in Medicine and Health, Macau University of Science and Technology, Taipa, Macau, China; g School of Health Sciences and Sports, Macao Polytechnic Institute, Macao, China; University of Arizona

**Keywords:** crystal structure, native human monoclonal antibody, RBS-C, S-RBD, SARS-CoV-2, therapeutic antibody

## Abstract

The emerging new lineages of severe acute respiratory syndrome coronavirus-2 (SARS-CoV-2) have marked a new phase of coronavirus disease 2019 (COVID-19). Understanding the recognition mechanisms of potent neutralizing monoclonal antibodies (NAbs) against the spike protein is pivotal for developing new vaccines and antibody drugs. Here, we isolated several monoclonal antibodies (MAbs) against the SARS-CoV-2 spike protein receptor-binding domain (S-RBD) from the B cell receptor repertoires of a SARS-CoV-2 convalescent. Among these MAbs, the antibody nCoV617 demonstrates the most potent neutralizing activity against authentic SARS-CoV-2 infection, as well as prophylactic and therapeutic efficacies against the human angiotensin-converting enzyme 2 (ACE2) transgenic mouse model *in vivo*. The crystal structure of S-RBD in complex with nCoV617 reveals that nCoV617 mainly binds to the back of the “ridge” of RBD and shares limited binding residues with ACE2. Under the background of the S-trimer model, it potentially binds to both “up” and “down” conformations of S-RBD. *In vitro* mutagenesis assays show that mutant residues found in the emerging new lineage B.1.1.7 of SARS-CoV-2 do not affect nCoV617 binding to the S-RBD. These results provide a new human-sourced neutralizing antibody against the S-RBD and assist vaccine development.

**IMPORTANCE** COVID-19 is a respiratory disease caused by severe acute respiratory syndrome coronavirus 2 (SARS-CoV-2). The COVID-19 pandemic has posed a serious threat to global health and the economy, so it is necessary to find safe and effective antibody drugs and treatments. The receptor-binding domain (RBD) in the SARS-CoV-2 spike protein is responsible for binding to the angiotensin-converting enzyme 2 (ACE2) receptor. It contains a variety of dominant neutralizing epitopes and is an important antigen for the development of new coronavirus antibodies. The significance of our research lies in the determination of new epitopes, the discovery of antibodies against RBD, and the evaluation of the antibodies’ neutralizing effect. The identified antibodies here may be drug candidates for the development of clinical interventions for SARS-CoV-2.

## INTRODUCTION

The outbreak of coronavirus disease (COVID-19), caused by severe acute respiratory syndrome coronavirus 2 (SARS-CoV-2) has led to over 200 million human infections and 4.2 million deaths worldwide as of early August 2021 (https://covid19.who.int). The passive administration of convalescent plasma is advocated for the early treatment of COVID-19 patients. Although some studies have suggested modest clinical benefits and measurable surrogate outcomes (1–3), others have provided weak evidence of clinical efficacy, probably due to the heterogeneous antibody content of convalescent plasma (4–6). Recently, human monoclonal antibodies (MAbs) isolated from the B cell receptor repertoires of convalescent-phase samples have become promising approaches to develop neutralizing monoclonal antibodies (NAbs) against SARS-CoV-2, with the advantages of scalability and therapeutic effectiveness (7–10).

Among the structural proteins of SARS-CoV-2, the spike (S) protein is the main target of human NAbs. The S protein belongs to the class I trimeric fusion membrane protein and mediates virus entry via its receptor-binding domain (S-RBD) binding to the host receptor angiotensin-converting enzyme 2 (ACE2) (11–14). The S-RBD undergoes a spontaneous conformational change between the “up” and “down” statuses, where only the up conformation is bound by the host receptor ACE2 (11, 15, 16). A large number of human NAbs that target S-RBD have been isolated and characterized (10, 17–29). The most recently identified NAbs target the S-RBD with three major epitopes—a receptor binding site (termed RBS), a CR3022 cryptic site, and an S309 proteoglycan site (27). The current RBS epitopes overlap the ACE2-binding surface, subdividing into three subgroups (RBS-A, RBS-B, and RBS-C) with different approaching angles and various ridge recognition patterns on the S-RBD surface. The NAbs with the RBS-A subgroup adopt nearly identical approaching angles and recognize only the up status of S-RBD. The RBS-B subgroup of NAbs recognize F486 of the S-RBD via a pocket on the antibody paratope, with various approaching angles. The RBS-C subgroup of NAbs target the opposite side of the RBS ridge and share the fewest overlapping regions with the ACE2-binding site (10, 19, 21). Intriguingly, the RBS-C subgroup of NAbs can bind both the up and down conformations of the S-RBD (10, 19, 21).

The uncontrolled spread in human populations continually leads to an evolutionary arms race between virus and host immune systems. Although numerous efforts have been made to develop antibody-based vaccines and therapeutics, the emergence of new SARS-CoV-2 lineages has marked a new phase in this COVID-19 pandemic. The SARS-CoV-2 lineage B.1.1.7, also referred to as VOC 202012/01 or 20I/501Y.V1, is more efficiently transmitted than previously circulating virus lineages (30, 31) (first reported by the authorities of the United Kingdom; https://www.gov.uk/government/publications/investigation-of-novel-sars-cov-2-variant-variant-of-concern-20201201). In addition to the B.1.1.7 variant, notable variants include B.1.351 (501Y.V2) (32, 33), B.1.1.28 (34, 35) (P.1 or 501Y.V3) (https://virological.org/t/phylogenetic-relationship-of-sars-cov-2-sequences-from-amazonas-with-emerging-brazilian-variants-harboring-mutations-e484k-and-n501y-in-the-spike-protein/585), and B.1.617 (https://www.cnbc.com/2021/05/10/who-classifies-triple-mutant-covid-variant-from-india-as-global-health-risk-.html). Several reports have suggested that these variants contain multiple genetic mutations in the S-RBD and may alter the conformation of the S-RBD or reduce susceptibility to NAbs. For example, the N501Y variant in the B.1.1.7 lineage increases the protein’s binding affinity to host receptor ACE2 (36). The N439K mutation is associated with escape from MAbs and serum-mediated neutralization (37). Some natural SARS-CoV-2 variants in S-RBD (including K417N, E484K, N501Y, A475V, L452R, V483A, F490L, and H519P) have been shown to have altered antigenicity (33, 38). Based on the structural information, the potential escaped mutants seem to affect the NAbs targeting the RBS-A and RBS-B sites of S-RBD more than the NAbs targeting RBS-C because of the differences of overlapping key residues with the ACE2-binding site. However, only five kinds of RBS-C subgroup NAbs have been found to neutralize the virus so far, and more antibodies of this subgroup urgently need to be identified.

Here, we isolated several effective NAbs against SARS-CoV-2 S-RBD derived from convalescent plasma. By determining the crystal structure of the human neutralizing antibody nCoV617 Fab fragment in complex with the SARS-CoV-2 S-RBD, we identified that nCoV617 is an RBS-C subgroup type NAb that specifically targets the SARS-CoV-2 S-RBD independent of mutations at residues F486 and N501 and some natural mutations in recently emerged SARS-CoV-2 lineages. Our work has provided an additional neutralizing antibody into S-RBD-specific NAb repertoires, which may benefit vaccines and therapeutic applications against the emerging SARS-CoV-2 lineages.

## RESULTS

### Isolation of S protein-specific human monoclonal antibodies.

**FIG 1:**
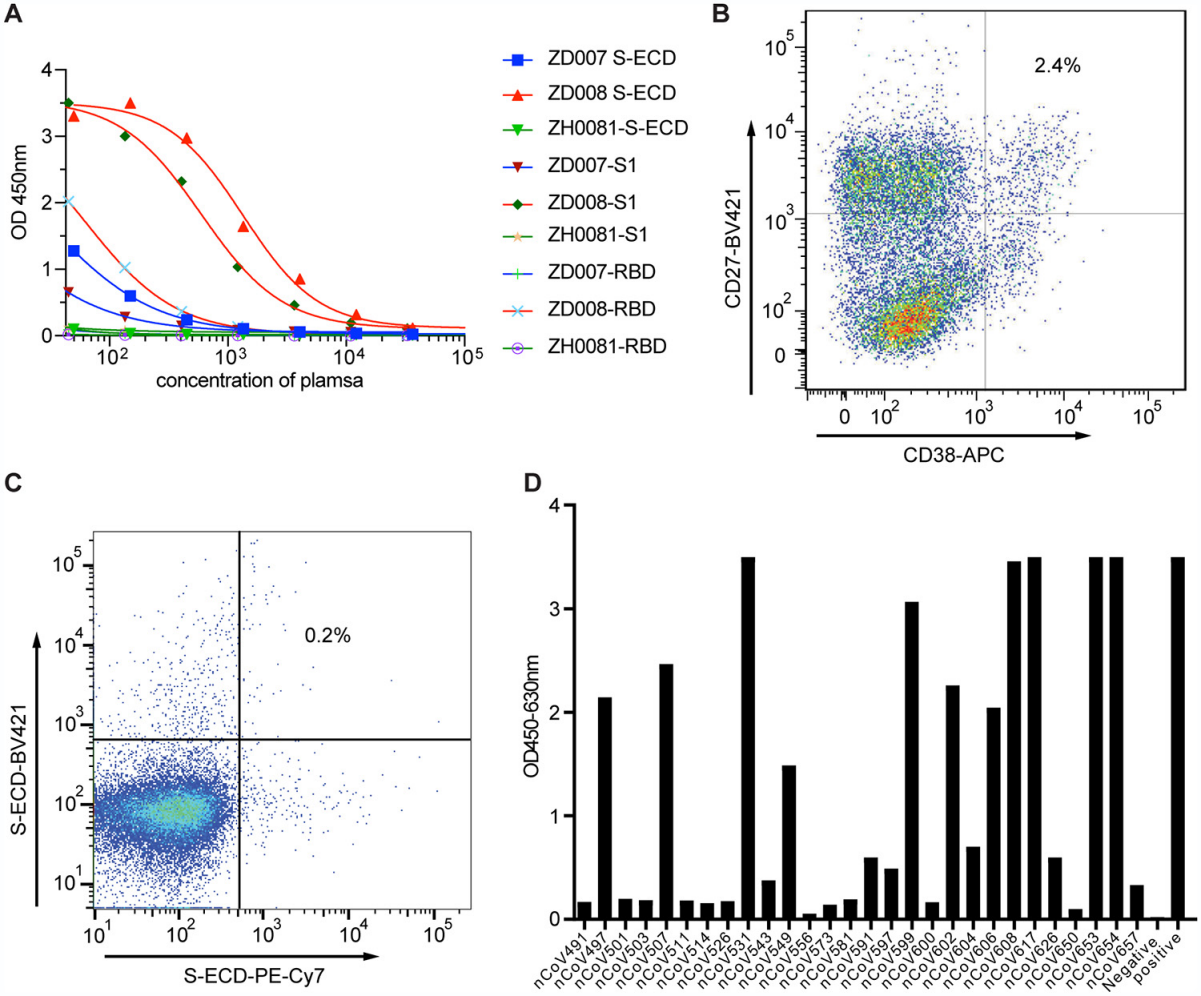
Acquisition and efficient identification of SARS-CoV-2 S antibodies. (A) Plasma antibody screening on enzyme-linked immunosorbent assay (ELISA) plates coated with recombinant S-ECD protein, S1 protein, and RBD protein. (B and C) Sorting of single plasma cells with CD38 and CD27 double-positive B cells (B) and single S-ECD protein-specific memory B cells (C) by FACS. (D) Binding ability of MAb expression from linear expression cassettes. A total of 28 antibodies expressed in transfected 293 cells were evaluated for binding to SARS-CoV-2 S-ECD. An irrelevant mAb (Ag005) and plasma of CoV008 were used as the positive control and negative control in panels A and D. All samples were performed in triplicate, and the means are presented.

To identify S protein-directed human neutralizing antibodies, we collected two longitudinal convalescent blood samples from a recovered patient at 17 and 25 days after the onset of the disease symptoms. The patient recovered from COVID-19 during the outbreak in Zhuhai, Guangdong Province, China. Plasma samples and peripheral blood mononuclear cells (PBMCs) were isolated for serological analysis and antibody isolation. Serum antibody titers to SARS-CoV-2 recombinant S truncated proteins (including the extracellular domain of S protein [residues 1 to 1,142, S-ECD], the S1 subunit [residues 1 to 678, S1], and the receptor-binding domain [residues 319 to 533, S-RBD]) were measured by enzyme-linked immunosorbent assays (ELISA). The serologic analysis demonstrated that serum antibody titers to the different versions of S protein were substantially high in the serum (Fig. 1A).

To take advantage of the high percentage of antigen-specific plasma cells, single plasma cells (Fig. 1B) with the phenotype CD3^−^/CD14-/CD16^−^/CD235a-/CD19^+^/CD20^low-neg^/CD27^hi^/CD38^hi^, as well as antigen-specific memory B cells with the phenotypes CD20^+^/CD27^+^ and S-ECD-BV421^+^/S-ECD-PE-Cy7^+^ (Fig. 1C), were sorted from PBMCs by fluorescence-activating cell sorter (FACS). The variable region of immunoglobulin (Ig) heavy- and light-chain gene segment (V_H_ and V_L_, respectively) pairs from the single sorted B cells were amplified by reverse transcriptase PCR (RT-PCR), sequenced, annotated, and expressed as recombinant MAbs using methods described previously (39). Recombinant MAbs were screened against SARS-CoV-2 recombinant S-ECD protein. In total, we identified 28 MAbs that reacted with SARS-CoV-2 recombinant S-ECD protein, including 19 MAbs from plasmocytes and 9 MAbs from memory B cells (Fig. 1D).

We found that IgG1 was the predominant isotype, at 64.3%, followed by IgA1 (17.9%), IgG2 (10.7%), IgG4 (3.6%), and IgM (3.6%) (see Table S1 in the supplemental material). V_H_ gene family usage in SARS-COV2 S protein-reactive antibodies was 21.4% V_H_1, 57.1% V_H_3, 14.3% V_H_4, 3.6% V_H_5, and 3.6% V_H_6 (Table S1), which was similar to the distribution of V_H_ families collected in the NCBI database. Out of 28 SARS-CoV-2 S protein-reactive antibodies, 4 had no mutation from their germ line V_H_ segments (Table S1). The chains of nCoV617 are encoded by the immunoglobulin (IG) genes IGHV3-30, IGHD3-3, IGHJ4, and IGKV1-44. IGHV of nCoV617 is 1.04% somatically mutated in its nucleotide sequence from the germ line gene, whereas its IGKV is 0.7% somatically mutated.

### The binding and neutralizing characteristics of isolated S protein-specific MAbs.

**FIG 2:**
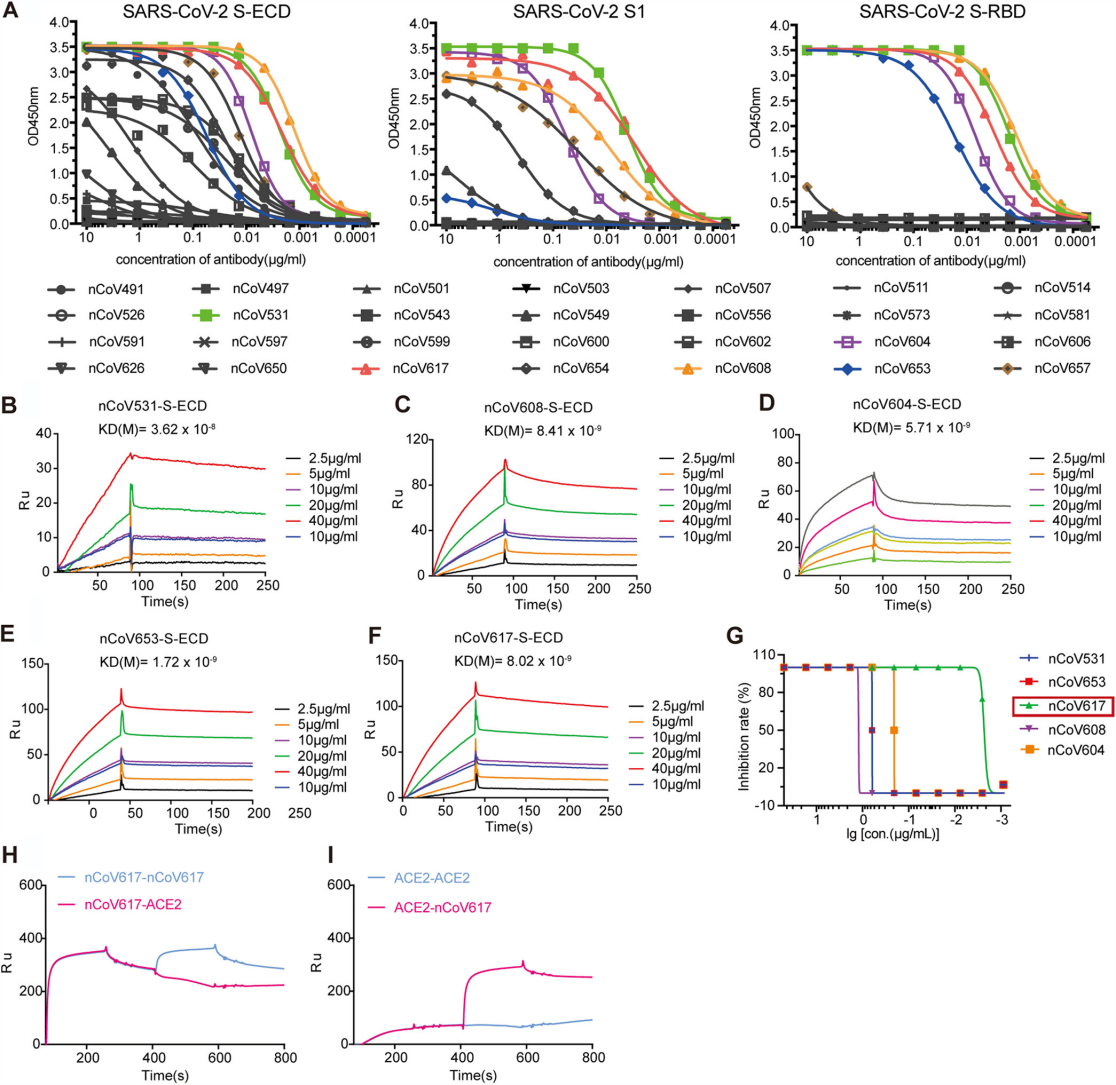
Affinity specificity and neutralizing abilities of the SARS-CoV-2 S protein MAbs. (A) ELISA analysis of 28 purified antibodies to S-ECD, S1, and S-RBD proteins. The concentration gradient of MAbs is the *x* axis, and the optical absorption values of OD_450_ are the *y* axis. The antibodies with five colored curves indicate that the antibodies have good combination ability with RBD. All samples were performed in triplicates, and the means are presented. (B to F) The binding affinity of five selected antibodies to the S-ECD protein was measured by SPR with a 1:1 binding model. *K_D_* values are shown above the individual plots. (G) The authentic virus neutralization ability of five selected antibodies. The neutralization activity curve of five monoclonal antibodies against Chinese endemic strains is shown. (H and I) Neutralization antibody nCoV617 and ACE2 competition for binding to RBD measured by SPR.

To screen potent antibodies against SARS-CoV-2 S proteins, we employed binding assays by using antibody dilutions on ELISA plates coated with either recombinant trimeric spike protein ECD, spike protein S1 subunit, or recombinant S-RBD (Fig. 2A). The MAbs nCoV531 (green curves), nCoV608 (orange curves), nCoV617 (red curves), and nCoV653 (blue curves) showed relatively stable binding. Dissociation constants with the SARS-CoV-2 S-RBD were obtained by surface plasmon resonance (SPR). For the above-described antibodies, the dissociation constant (*K_D_*) ranged from 1.72 to 36.2 nM (Fig. 2B to F).

Authentic virus neutralization assay results showed that among the five antibodies that were screened, nCoV617 had the most potent inhibitory ability, with a 90% inhibitory efficiency at 0.01 μg/ml (Fig. 2G). Next, we examined the potential competitive binding of nCoV617 and ACE2 with SARS-CoV-2 S-RBD. The recombinant SARS-CoV-2 S-RBD is covalently immobilized on the CM5 sensor chip and saturated with nCoV617 or ACE2, and soluble nCoV617 or ACE2 is injected and flows through the CM5 sensor. As shown in Fig. 2H and I, the S-RBD-binding capacity of ACE2 is strongly competitive by nCoV617. Taken together, these results indicate that the antibody nCoV617 is a potent neutralizing antibody to SARS-CoV-2, with classic receptor-binding site (RBS) epitopes competing with ACE2 binding.

### nCoV617 can effectively reduce the viral load and lung damage in mice.

**FIG 3:**
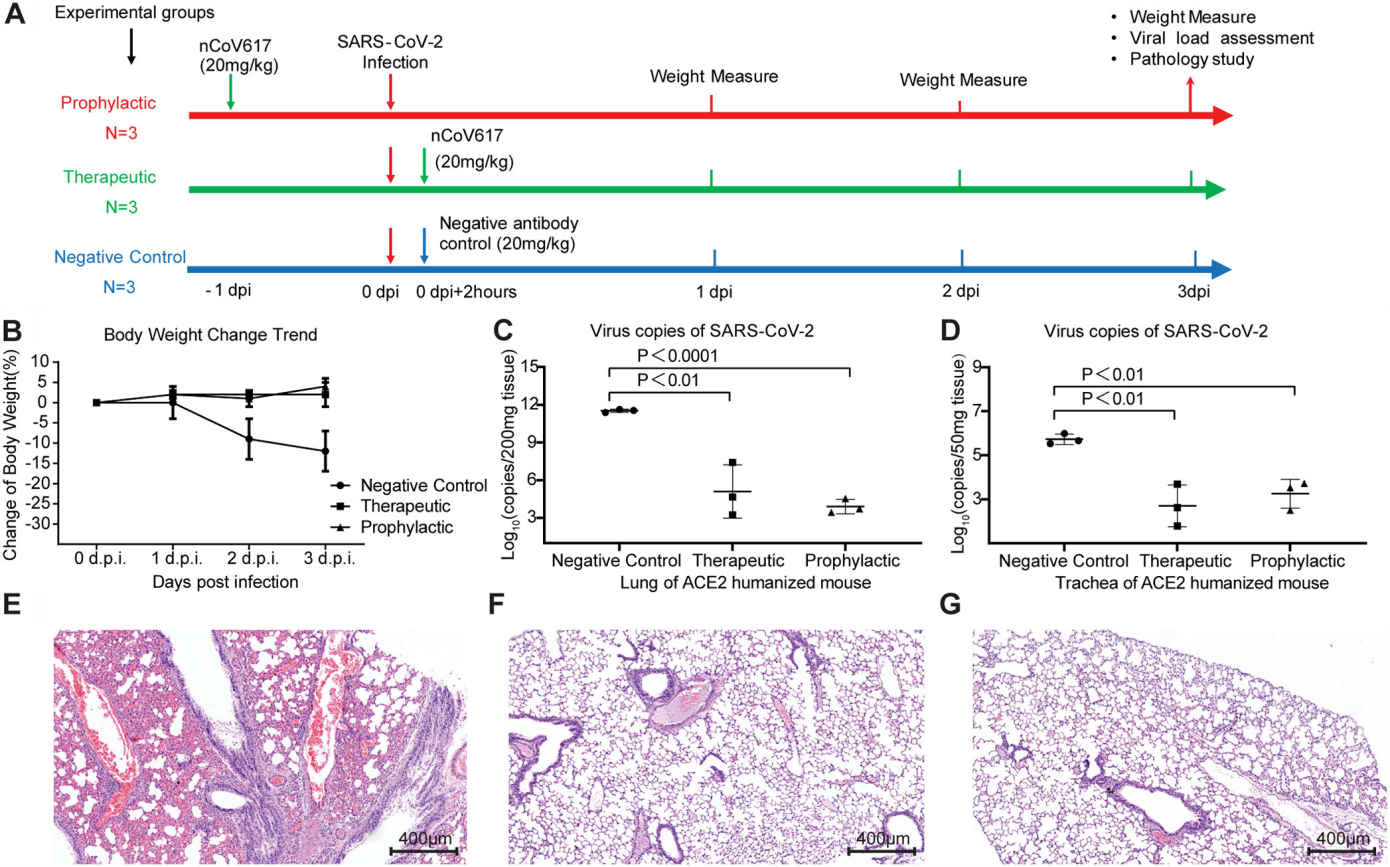
The protection efficiency of the neutralizing antibody nCoV617 in the ACE2-HU mouse model postinfection with SARS-CoV-2 (a Chinese endemic strain). (A) The scheme flowchart of the experiment. (B to D) In the ACE2 humanized mouse model, body weight (B) and virus copies of SARS-CoV-2 in lung (C) and in trachea (D) count in the therapeutic, prophylactic, and irrelevant MAb (Ag005) negative-control groups. (E to G) Hematoxylin-eosin (HE) staining of ACE2-HU mouse lung tissue sections of the negative-control (E), prophylactic (F), and therapeutic (G) groups. Results presented in panels B to D are representatives of three independent biological experiments.

We further tested nCoV617 *in vivo* in SARS-CoV-2-infected ACE2-HU mice (a southern model organism) in a preventive and therapeutic environment. SARS-CoV-2 (a Chinese epidemic strain) incubated intratracheally in mice (6 to 8 weeks old) was challenged with a 1 × 10^4^ 50% tissue culture infection dose (TCID_50_). In the treatment group, nCoV617 (20 mg/kg) was injected intravenously 2 h postinfection. The control group (*n* = 3) was given the irrelevant IgG1 antibody, Ag005 (20 mg/kg), 2 h after infection, and the prevention group was given the same standard nCoV617 24 h before infection (Fig. 3A). After challenge, the mice were weighed every day, and the lungs and trachea of the mice were collected at 3 days postinfection (dpi). The weight of the mice in the control group decreased every day from the first day after infection (Fig. 3B). In contrast, the nCoV617 treatment group and the prevention group maintained the weight of the mice unchanged after administration (Fig. 3B). Evaluation of the virus titers in the lungs and trachea found that, compared with the control group, nCoV617 reduced the virus titers in the lungs by approximately 6∗log10 of copies in 200 mg tissue and approximately 3∗log10 of copies in 200 mg tissue in the trachea by 3 dpi (Fig. 3C and D). In addition, in the prevention group of three mice, a single dose of nCoV617 (20 mg/kg) before challenge with SARS-CoV-2 protected mice from SARS-CoV-2 infection. The lowest level of virus was detected in the lungs of this group. Compared with the control group, the viral load in the trachea was significantly lower, similar to that of the treatment group (Fig. 3C and D). These results indicate that the nCoV617 antibody has a powerful ability to prevent SARS-CoV-2 infection.

In addition to reducing the virus titer, we also investigated whether nCoV617 also inhibited pathological lung injury in mice challenged with SARS-CoV-2. One mouse in each group was euthanized and necropsied at 3 dpi. Control mice showed interstitial pneumonia, which was characterized by thickening of the alveolar septum and proliferation and fibrosis of fibroblasts, as well as intense infiltration of monocytes and lymphocytes. In some alveolar cavities, cellulose exudation was observed to form a hyaline membrane and pulmonary hemorrhage. There was also apparent thrombosis in the pulmonary capillary lumen, bronchiole necrosis, and the accumulation of shed epithelial cells (Fig. 3E). In contrast, after prophylaxis or treatment, mice showed limited pathological lung damage. Compared with the control mice, the autopsied mice from the treatment group and the prevention group had complete alveolar structure, reduced edema and hyaline membrane formation, less fibrosis, and less leukocyte infiltration (Fig. 3F and G). In addition, no severe bronchial or pulmonary capillary lesions were observed (Fig. 3F and G). Therefore, in terms of prevention and treatment, nCoV617 inhibits the SARS-CoV-2 virus titer and reduces infection-related lung damage.

### Crystal structure of MAb nCoV617 in complex with S-RBD.

To explore the molecular recognition mechanism of the MAb nCoV617 with S-RBD, we next solved the complex structure at 2.5-Å resolution by X-ray crystallography. Briefly, the complex structure was determined by molecular replacement using the S-RBD structure (PDB: 6M0J) and monoclonal antibody C135 (PDB: 7K8Z) as the search models. The asymmetric unit of the crystal contains only one complex of SARS-CoV-2 S-RBD with nCoV617. The complete statistics for data collection, phasing, and refinement are presented in Table S2. The complex structure is deposited in the Protein Data Bank under the access code 7E3O.

**FIG 4:**
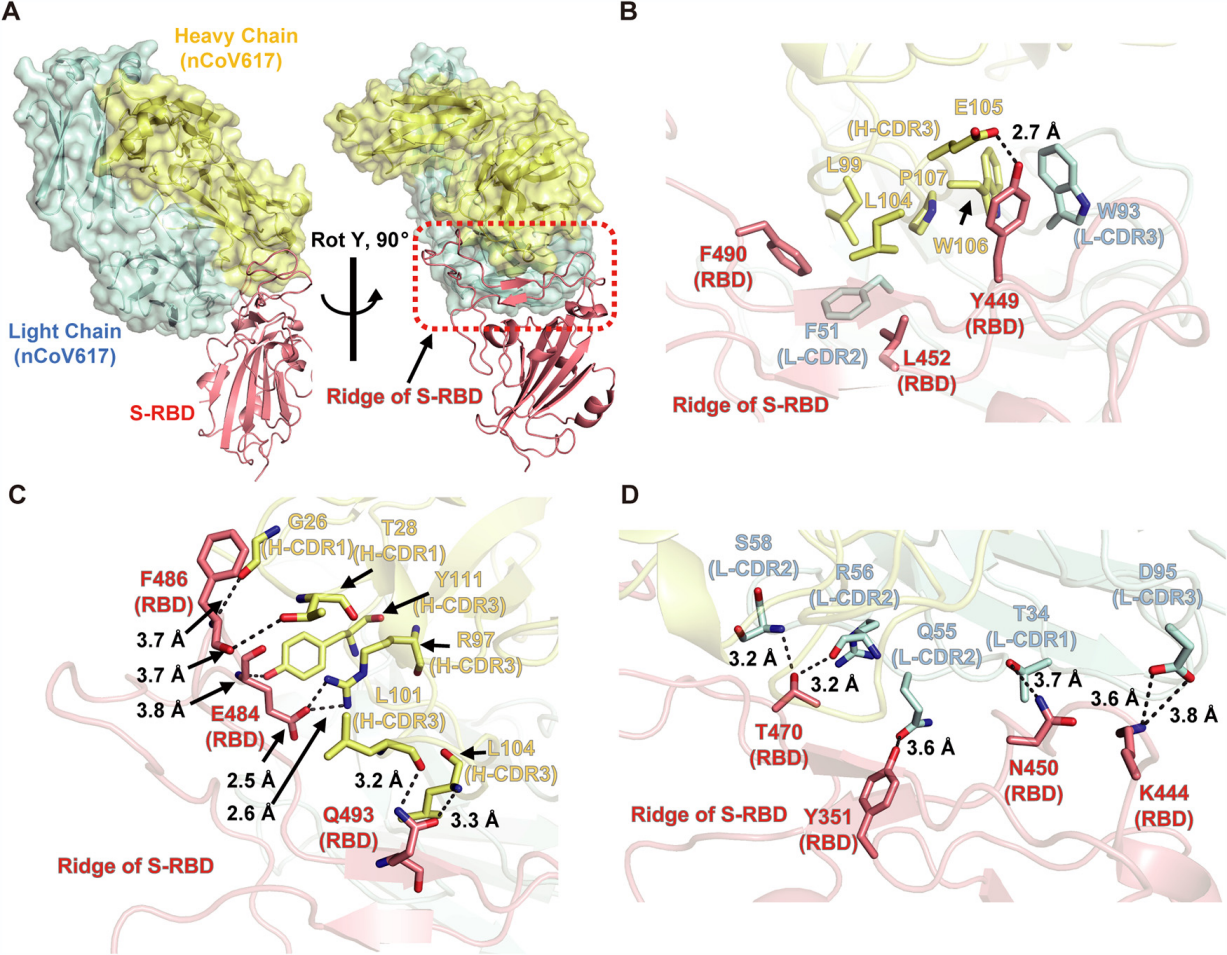
Complex structure of MAb nCoV617 with SARS-CoV-2 S-RBD. (A) Overall structure of the MAb nCoV617-SARS-CoV-2 S-RBD complex with orthogonal angles. The light chain (cyan) and heavy chain (yellow) of MAb nCoV617 are illustrated with the ribbon and surface. SARS-CoV-2 S-RBD is illustrated with the ribbon, and the red dashed frame area is the ridge of RBD. (B) The Y449, L452, and F490 residues of S-RBD interact with the hydrophobic and strong hydrogen bonds of MAb nCoV617. (C) The hydrogen bond between the end of the S-RBD ridge region interacts with H-CDR1 and H-CDR3 of nCoV617 VH. (D) The long-distance hydrogen bonding interaction between S-RBD and the V_L_ of nCoV617.

The overall structure demonstrated that complementarity determining regions (CDRs), including H-CDR1, H-CDR3, L-CDR1, L-CDR2, and L-CDR3, of nCoV617 recognize the ridge region of S-RBD with an interface area of 672.2 Å^2^ to V_H_ and 446.2 Å^2^ to V_L_, respectively (Fig. 4A and Fig. S1). With the buried surface criterion (BSA, >10 Å^2^), 10 residues of the RBD were involved in the interaction with nCoV617 by analysis with the PISA server (https://www.ebi.ac.uk/pdbe/pisa/). Briefly, the Y449, L452, and F490 residues of S-RBD are located in the adjacent area of the ridge two-strand antiparallel β-sheet, interacting with both chains of the NAb nCoV617 mainly via a hydrophobic network (Fig. 4B). Additionally, the tip of the ridge region of S-RBD forms multiple hydrogen bonds (H-bonds) with residues from H-CDR1 and H-CDR3 of nCoV617 V_H_. Among these interactions, backbones of F486 (S-RBD) form weak interactions with G26 and T28 from H-CDR1, while the δ-amide group of Q493 (S-RBD) is bound by the backbone of L101 and L104 from H-CDR3. Notably, the δ-carboxy group of E484 (S-RBD) forms strong interactions with the ɛ-amide group of R97 from H-CDR3 (Fig. 4C). In contrast, the interactions between the V_L_ of nCoV617 and the S-RBD are mainly mediated by long-distance H-bonds (Fig. 4D). Taken together, nCoV617 primarily recognizes several critical epitopes on the ridge region of the S-RBD via various hydrophilic and hydrophobic interactions, excluding the Y501 residue found in the newly emerged SARS-CoV-2 strain B.1.1.7 (Fig. S1).

Previous evidence has suggested that the S-RBD fluctuates between up and down conformations in the context of the S trimer (16). In the S-RBD up conformations, the ridge region is completely exposed to the solvent region and accessible by nCoV617. To examine whether nCoV617 binds to the S-RBD down conformation, we next superimposed the nCoV617-S-RBD complex with trimeric S protein cryo-electron microscopy (cryo-EM) models (PDB: 6VXX and 7DF4). As shown in Fig. S2, alignment of the nCoV617-S-RBD complex structure into the “all-down” RBD conformation of the S-ECD cryo-EM structure (PDB: 6VXX) demonstrated that nCoV617 is tolerated to be fitted into the model without any clash (Fig. S2A). However, a slight clash to the up state S-RBD was observed when the nCoV617-S-RBD was aligned to the down RBD in the “one-up/two-down” S-ECD model (PDB: 7DF4) (Fig. S2B). These alignment results reflect that nCoV617 potentially binds to both up and down RBD states in the context of full-length S protein.

### The effect of S-RBD mutants on NAb nCoV617 recognition.

**FIG 5:**
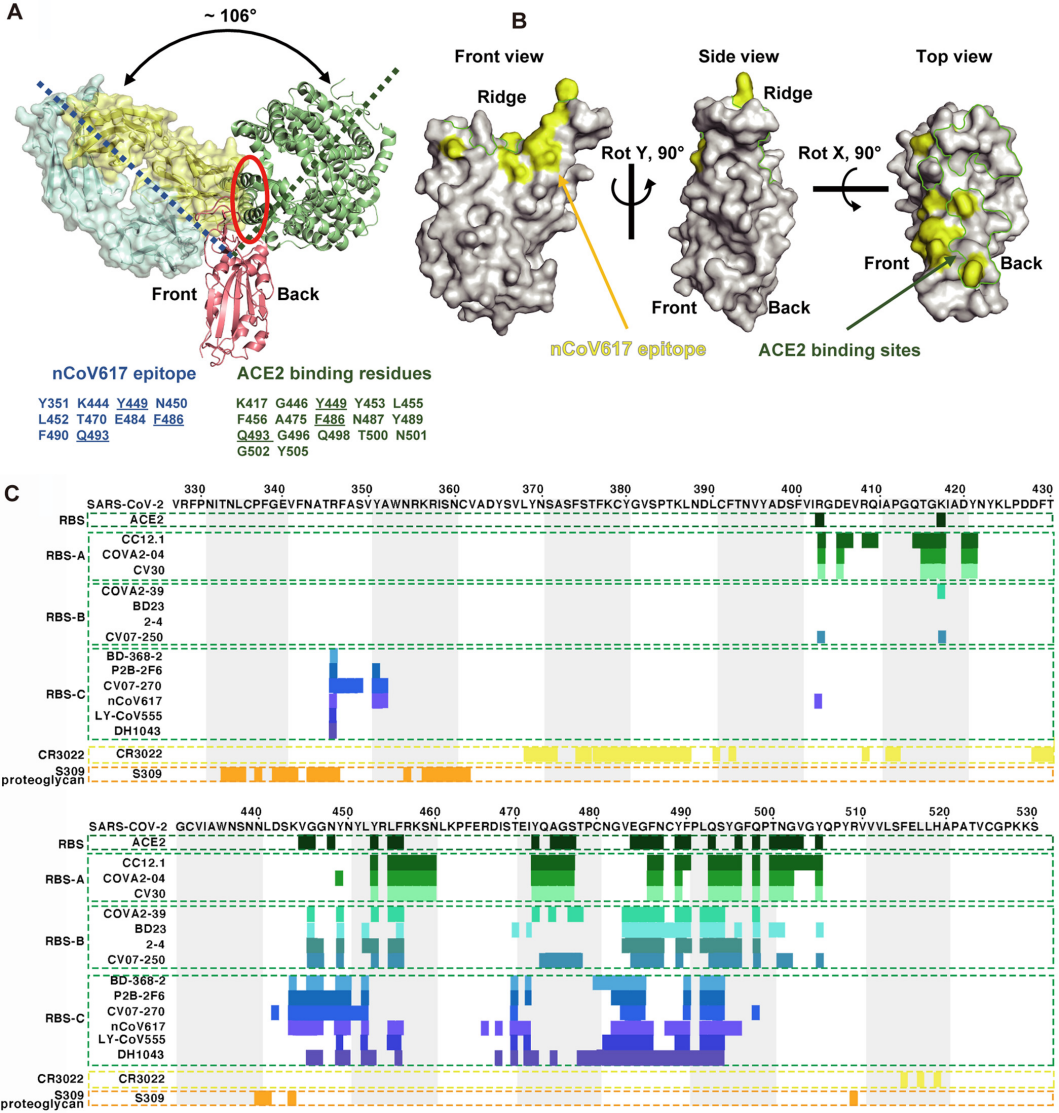
Epitope classification and comparison of MAb nCoV617. (A) Angles and clash between MAb nCoV617 and ACE2 (PDB: 6M0J) when they were binding SARS-CoV-2 S-RBD. The dashed blue and green lines indicate the long axes of nCoV617 Fab and ACE2, respectively, and the red solid ellipse indicates the clash between nCoV617 Fab and ACE2 in binding to the RBD. The nCoV617 Fab epitope and ACE2 binding sites are listed in the lower panel, and the binding residues shared between the Fab and ACE2 are underlined. (B) The footprint of nCoV617 Fab and ACE2 on SARS-CoV-2 RBD. Yellow and green represent the footprints of nCoV617 Fab and ACE2, respectively. (C) Epitope comparison between antibodies against SARS-CoV-2 S-RBD. Residue numbers corresponding to the SARS-CoV-2 RBD are labeled every 10 residues above the sequence panel. The antibody clusters that SARS-CoV-2 S-RBD binds to different epitopes are shown between the colored dotted lines, and the epitope residues are represented by colored bars under the sequence panel. The PISA program was used to analyze the interaction between the RBD and its ligands (including antibodies and ACE2). The RBS antibodies and their PDB IDs are CC12.1 (PDB ID: 6XC2), CV30 (PDB ID: 6XE1), COVA2-04 (PDB ID: 7JMO), COVA2-39 (PDB ID: 7JMP), BD-236 (PDB ID: 7CHB), 2-4 (PDB ID: 6XEY), CV07-250 (PDB ID: 6XKQ), BD-368-2 (PDB ID: 7CHE), P2B-2F6 (PDB ID: 7BWJ), CV07-270 (PDB ID: 6XKP), and 6W41, 6WPS, 6M0J for CR3022, S309, and ACE2 in complex with RBD. The analysis here uses the buried surface (BSA, >0Å^2^) as the standard instead of using contact residues to define the epitope. ACE2-binding residues are shown in dark green. The epitope of the RBS antibody is displayed in various shades of green and blue, the epitope of the MAb nCoV617 is displayed in light purple, the CR3022 concealed site antibody is displayed in yellow, and the S309 proteoglycan site antibody is displayed in orange.

To examine the capacity of nCoV617 to block the interaction between S-RBD and ACE2, the nCoV617-S-RBD complex structure was superimposed with the S-RBD-ACE2 complex structure. As shown in Fig. 5A, the approaching angle of nCoV617 is dramatically different from that of ACE2, with an approximately 106° deviation measured by the Cα atoms of residues E214 (nCoV617-V_L_), Y495 (S-RBD), and P258 (ACE2). However, the V_H_ of nCoV617 sterically blocked ACE2 binding, mediated by overlapping residues Y449, F486, and Q493 shared with ACE2 binding sites (Fig. 5A). Notably, the ridge region of the S-RBD interacts with nCoV617 mainly on the front side, whereas ACE2 binds to its back side (Fig. 5A). To explore the spatial relationship of the epitope sites, the nCoV617 epitope is highlighted along with the ACE2 binding sites. As shown in Fig. 5B, only limited overlapping residues were found at the interacting surfaces. As a result, the total volume of clashed residues was only ∼1,026 Å^3^, much less than the previously reported RBS-A and RBS-B subgroup antibodies (Fig. 5B). Subsequently, epitope alignment suggested that the nCoV617 epitope belongs to the RBS-C subgroup (Fig. 5C).

**FIG 6:**
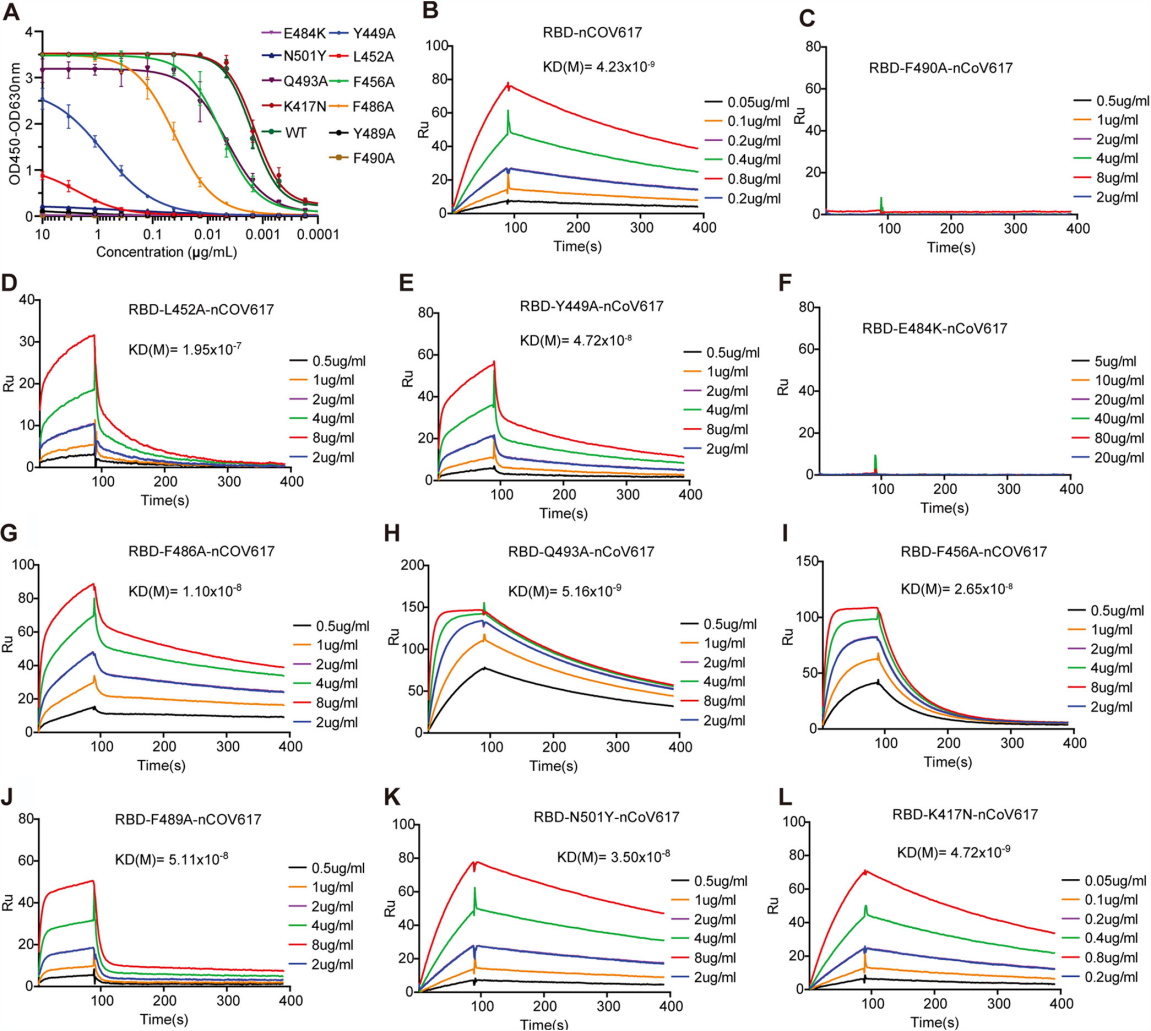
Impact of key SARS-CoV-2 S-RBD residues on MAb nCoV617 binding. (A) The nCoV617 was analyzed for binding to RBDs with different mutation residues measured by ELISA. (B to L) The binding affinity of SARS-CoV-2 S-RBD wild-type (B) and nine mutations (F490A [C], L452A [D], Y449A [E], E484K [F], F486A [G], Q493A [H], F456A [I], F489A [J], N501Y [K], and K417N [L]) to nCoV617 was measured by SPR. *K_D_* values are shown above the individual plots.

To assess the structural basis of the nCoV617-S-RBD complex and whether natural mutants affect the antibody-binding affinity, we next conducted single-site alanine mutagenesis SPR assays for the key residues in the S-RBD. Among the hydrophobic interaction residues, the F490A mutations resulted in complete loss in the binding of nCoV617 compared with wild-type S-RBD (with a *K_D_* value of 4.23 nM; Fig. 6A to C). Meanwhile, the other hydrophobic interaction residue mutant, L452A, showed an approximately 50-fold reduction (with a *K_D_* value of 195 nM; Fig. 6D). The remaining mutant in this interaction network, Y449A, resulted in a moderate reduction (with a *K_D_* value of 47.2 nM; Fig. 6E). The V_H_ recognition mode suggested that E484 is pivotal in nCoV617 binding, and F486 and Q493 show weak binding to the NAb. As expected, the E484K mutant on the V_H_ recognition surface resulted in complete loss of nCoV617 binding (Fig. 6F), whereas the natural F486A and Q493A mutants only slightly affected the binding (with a *K_D_* value of 11.0 nM and 5.16 nM; Fig. 6G and H). Furthermore, the unbound residue mutants F456A and F489A were slightly affected by nCoV617 binding (with *K_D_* values of 26.5 nM and 51.1 nM, respectively; Fig. 6I and J). It is worth noting that the newly emerged mutations N501Y and K417N have almost negligible influence on the binding affinity of nCoV617 (with *K_D_* values of 35.0 nM and 4.72 nM, respectively; Fig. 6K and L).

In summary, the complex structure of nCoV617-S-RBD demonstrates that hydrophobic interactions and E484 hydrophilic interactions are the main driving forces.

## DISCUSSION

Human antibody isolation technology from convalescent individual B cells has been exploited to develop treatments against HIV, Ebola, and Middle Eastern respiratory syndrome (MERS) (39–41), as well as in this ongoing COVID-19 pandemic (10, 17–29). Although numerous NAbs have been developed against SARS-CoV-2, combinations of two or more NAbs are expected to provide more efficiency toward the emerging new lineage of SARS-COV-2. Here, we report the structural basis of the neutralizing activity NAb nCoV617 selected from 28 monoclonal antibodies isolated from individuals in the recovery period of COVID-19. We next provide the atomic details of the complex’s epitopes and paratopes via X-ray crystallography technology. Furthermore, comparing the approach angles and potential steric clashes with receptor ACE2 in binding to S-RBD, it was found that nCoV617 acts by interfering with the interaction with RBD and ACE2. Surprisingly, comparing nCoV617 with the reported epitopes of neutralizing antibodies, it was found that in most neutralizing antibodies that overlapped with ACE2 epitopes, there were only three shared epitope residues between nCoV617 and ACE2, which belonged to the RBS-C class. Among the RBS-C class antibodies, the approach angle between nCoV617 and ACE2 is the largest. Animal experiments have shown that nCoV617 reduced the virus titer in mice, significantly improved the weight loss caused by virus infection, and alleviated the symptoms in both preinfection prevention and postinfection treatment. This suggests that nCoV617 has the potential to be a promising drug candidate for the prevention and treatment of COVID-19.

Further *in vitro* binding assays confirmed that the recognition of nCoV617 by the S-RBD was mainly driven by hydrophobic interactions and E484 hydrophilic interactions. Interestingly, these interactions do not involve several newly emerging SARS-CoV-2 strains, suggesting that nCoV617 may serve as a potent neutralizing antibody to these strains. With the help of structural information, it is found that the S-RBD variants N501Y and K417N in the recently reported British mutant SARS-CoV-2 B.1.1.7 are not on the binding interface of nCoV617 and have very little effect on the binding affinity of nCoV617. It can be reasonably speculated that the N501Y and K417N mutations do not affect the binding energy of the antibody. Notably, the NAb nCoV617 ultimately lost binding to the S-RBD bearing the E484 mutant. The other recently emerged SARS-CoV-2 lineages include the B.1.351 (501Y. V2) and B.1.1.28 (P.1 or 501Y. V3), which both acquire the E484K mutation in S-RBD, and the B.1.617, which acquires the E484Q mutation in S-RBD. Therefore, nCoV617 may not be effective against viruses bearing the E484 mutation in the S-RBD region. Additionally, the F490A mutant also completely loses the ability of nCoV617 to bind to S-RBD. However, a mutant strain containing F490A has not yet been selected *in vitro*. This implies that the NAb nCoV617 may not work as expected, and further research is needed for these new variants. However, it is worth noting that as of 29 June 2021, the SARS-CoV-2 variants of concern (VOCs) updated by the WHO only includes 501Y and 478K mutations, excluding 484K.

At present, although a vaccine against COVID-19 has been implemented, its effective period and side effects remain unknown. In addition, since the initial outbreak of COVID-19, the SARS-CoV-2 S protein has continuously mutated to adapt to the environment and enhance its ability to escape the immune system, especially the S-RBD. Therefore, a single antibody may not be sufficient to address such rapid mutations. The combination of multiple neutralizing antibodies may become an effective and feasible method for antibody therapy and prevention. Our results have revealed the neutralizing mechanism of the NAb nCoV617, which may benefit both prophylactic and therapeutic interventions for the newly emerging SARS-CoV-2 lineage B.1.1.7 and provide effective options for comprehensive antibody therapy, but for the lineages B.1.351, B.1.1.28, B.1.617, and B.1.617.2, the effect of nCoV617 may be greatly reduced. The influence of these new lineages on the neutralization of nCoV617 needs further verification.

## MATERIALS AND METHODS

### Recombinant SARS-CoV-2 S-ECD, S1, and S-RBD proteins and human ACE2.

For cocrystallization, recombinant SARS-CoV-2 S-RBD (residues 319 to 527) was expressed using the Bac-to-Bac baculovirus system. Specifically, the SARS-CoV-2 RBD containing the gp67 secretion signal peptide and a C-terminal octahistidine was inserted into pFastBac-HTB vectors and transformed into DH10Bac component cells. The recombinant bacmid was extracted and further transfected into Sf9 cells using Cellfectin II reagents (Invitrogen). The recombinant viruses were harvested from the transfected supernatant and amplified to generate a high-titer virus stock. Viruses were then used to infect Sf9 cells for RBD expression. Briefly, secreted RBD was harvested from cell culture medium and purified sequentially on a Ni-NTA column and Superdex 200 size exclusion column (GE Healthcare) (42). For the enzyme-linked immunosorbent assay (ELISA), the recombinant SARS-CoV-2 spike protein (S-ECD, His and Flag tag ZO3481) and S1 protein (His tag Z03507) were purchased from GenScript. S-RBD protein (amino acids [aa] 319 to 533) with a 6 histidine tag at the C-terminal end was designed and codon-optimized for Sf9 cell expression. Expression and purification of the human angiotensin-converting enzyme ectodomain (ACE2, residues 1 to 614) (GenBank accession no. NM_001389402.1) fused to the Fc region of human IgG (hFc) was performed as previously described (43), and cleavage of the Fc fragment was carried out with thrombin (15).

### Fluorescence-activated cell sorting of single plasma cells and memory B cells.

Blood samples were collected 17 and 25 days after the onset of the disease from a patient who recovered from COVID-19 infection. Peripheral blood mononuclear cells (PBMCs) and plasma were isolated from blood by Ficoll-Paque PLUS (GE, 17-1440-02) density gradient centrifugation. All work related to human subjects was carried out in compliance with institutional review board protocols approved by the institutional review board of the Fifth Affiliated Hospital of Sun Yat-sen University. Single plasma cells with the surface markers CD3^−^, CD14^−^, CD16^−^, CD235a^−^, CD19^+^, CD20^low-neg^, CD27^hi^, and CD38^hi^ and memory B cells with the surface markers CD3^−^, CD14^−^, CD16^−^, CD235a^−^, CD20^+^, CD19^+^, CD27^+^, and SARS-CoV-2 S-ECD-BV421^+^/S-ECD-PE-Cy7^+^ (BD Biosciences and Invitrogen) were sorted into individual wells in 96-well microtiter plates containing cell lysis and RT buffer for Ig gene amplification by fluorescence activated cell sorting (FACS) as previously described (44) on a BD FACS Aria SORP. Data were analyzed using BD FACS Diva 8.0.1 software.

### Isolation and expression of Ig V_H_DJ_H_ and V_L_ genes.

Genes encoding the variable region of Ig heavy and light chains (*V_H_DJ_H_* and *V_L_*) were amplified by reverse transcription (RT) and nested PCR using a previously described method (45). PCR products of Ig *V_H_DJ_H_* and *V_L_* genes were purified using a PCR purification kit (Qiagen), sequenced in forward and reverse directions (Thermo Fisher Scientific) and annotated by using IMGT/V QUEST (www.imgt.org/IMGT_vquest). Functional *V_H_DJ_H_* and *V_L_* genes were used for assembling full-length Ig heavy- and light-chain linear expression cassettes by one-step overlapping PCR (45). HEK-293T cells in 12-well plates were transfected with the assembled Ig heavy- and light-chain pairs derived from the same single individual plasma cells using Effectene (Qiagen) as the transfection reagent (45).

### Production of recombinant IgG and Fab antibodies.

For production of purified full-length IgG1 antibodies, the *V_H_DJ_H_* and *V_L_* genes were cloned into the pCDNA3.1^+^ (Invitrogen) mammalian expression vector containing either the human IgG1 constant region gene, the human lambda light-chain constant region, or the lambda light-chain constant region gene using standard recombinant DNA technology (45). For the production of purified nCoV617Fab antibody, a stop codon TGA was introduced after the sequence (5′-TCTTGTGACAAA-3′) encoding amino acid residues, SCDK, just before the hinge of the human IgG1 constant region (46). Recombinant IgG1 antibodies and the nCoV617Fab antibody were produced in 293F cells cultured in serum-free medium by cotransfection with the generated full-length IgG1 or Fab heavy- and light-chain gene expression plasmid pairs using polyethylenimine (47). Full-length IgG1 antibodies were purified by using protein A column chromatography as described previously (45). The nCoV617Fab antibody used for the crystal structure was purified by Lambda FabSelect, an affinity resin designed for the purification of human Fab with a lambda light chain (GE Healthcare) (46).

### Analysis of the binding of plasma antibodies and isolated MAbs to the S-ECD, S1, and RBD proteins by ELISA.

We collected plasma from a patient at 17 and 25 days after the onset of the disease symptoms and measured plasma antibody titers using recombinant SARS-CoV-2 S-ECD, S1, and S-RBD proteins as antigens to coat ELISA plates. Antibodies in the supernatant of the transfected HEK293T cultures harvested 3 days after transfection were screened by ELISA as described previously (45). The binding of purified antibodies to the S-ECD, S1, or S-RBD protein was also analyzed by ELISA. Briefly, all protein antigens (S-ECD, S1, and S-RBD) were used at 200 ng/well to coat 96-well high-binding ELISA plates (Nunc 442404) using carbonate-bicarbonate buffer at pH 9.6. Plates were incubated overnight at 4°C and blocked at room temperature for 2 h with phosphate-buffered saline (PBS) blocking buffer containing 5% wt/vol goat serum and 0.05% Tween 20. Plasma or supernatant of transfected 293T cell cultures or purified MAbs in serial dilutions in PBS were incubated at 37°C for 1 h. The secondary antibody, goat-anti-human IgG-HRP (1:10,000 dilution) (Promega, W4031), diluted in blocking buffer was added and incubated at 37°C for 1 h. These plates were then washed 5 times with PBS and developed with 100 μl/well tetramethylbenzidine (TMB) substrate (Solarbio PR1200). The reaction of the mixtures in the plates was stopped with 50 μl/well 2 M H_2_SO_4_ and read at a wavelength of an optical density at 450 nm (OD_450_) by an ELISA reader.

### Affinity and kinetic measurements by SPR.

The binding affinity (*K_D_*), association rate (*k_a_*) and dissociation rate (*k_d_*) of purified MAbs to S-ECD or RBD protein were determined by SPR using a Biacore X100 System (GE Healthcare). Anti-human Fc IgG antibody was first immobilized on a CM5 chip to approximately 6,000 response units (RUs) by covalent amine coupling using a human antibody capture kit (GE Healthcare). Purified MAbs were captured on channel 2 of the CM5 chip to approximately 200 RUs. Five 2-fold serial dilutions of the S-ECD protein starting at 40 μg/ml were injected at a rate of 20 μl/min for 90 s with a 300-s dissociation. The chip was regenerated by injection of 3 M MgCl_2_ for 30 s. All experiments were performed at room temperature, and data were analyzed using Biacore X100 evaluation software (version 2.0.1). Curves were fitted to a 1:1 binding model to determine kinetic rate constants (*k_a_* and *k_d_*). *K_D_* values were calculated from these rate constants. To test the nCoV617 and ACE2 competition for binding to RBD, the RBD was covalently immobilized on a CM5 sensor chip and first saturated with antibody and then flowed through with soluble ACE2.

### Authentic SARS-CoV-2 neutralization assay in Vero cells.

Authentic neutralization assays were performed using Vero cells in 96-well cell culture plates (Corning; no. 3988). In the antibody screening assay, various concentrations of MAbs (3-fold serial dilution) were mixed with 50-μl 30-fold TCID_50_ authentic SARS-CoV-2 (Chinese endemic strains) and incubated at 37°C and 5% CO_2_ for 1 h. After incubation, 100 μl of Vero cell suspension (density, 1.5 to 2.5 × 10^4^ cells/ml) was added to the mixture for each well, and then the plates were placed in an incubator at 37°C with 5% CO_2_ for 3 to 4 days. All experiments were performed in a biosafety level 3 facility of the Institutional Biosafety Committee of the Institute of Medical Biology (IMB) in the Kunming National High-Level Biosafety Primate Research Center.

### Authentic SARS-CoV-2 neutralization assay in the ACE2-HU mouse model.

The human ACE2 gene transgenic mouse model in C57BL/6J mouse background was developed by the Shanghai Model Organisms Center, Inc. ACE2-HU mice, aged 6 to 8 weeks (southern model organism, female), were randomly divided into three groups—the prophylactic group, the therapeutic group, and the irrelevant negative-antibody group. The antibody was administered at an intramuscular dose of 20 mg/kg. The prophylactic group was given nCoV617 antibody 24 h before challenge, the therapeutic group was given nCoV617 antibody 2 h after challenge, and the negative-antibody group was given irrelevant IgG1 antibody Ag005 2 h after challenge. All animals were challenged by nasal infusion of 1 × 10^4^ TCID_50_ SARS-CoV-2 (Chinese epidemic strain; GenBank accession no. MT226610). The animals were weighed every day after challenge, and the lungs and trachea of the animals were collected on the third day after infection. Tissues were taken for paraffin embedding and sectioning, and sections were stained by hematoxylin-eosin staining. Total RNA of the lung and trachea was extracted with TRIzol universal total RNA extraction reagent (Tiangen; DP424), and the copy number of the SARS-CoV-2 gene was detected by one-step quantitative PCR using a real-time fluorescence quantitative PCR instrument (Bio-Rad; CFX96). Primers were as follows: ORF1ab-F, 5′-CCCTGTGGGTTTTACACTTA-3′; ORF1ab-R, 5′-ACGATTGTGCATCAGCTG-3′; and ORF1ab-P, 5′-CCGTCTGCGGTATGTGGAAAGGTTATGG-3′. All animal experiments were conducted with prior approval from the Animal Ethics Committee of the Institute of Medical Biology, IMBCAMS, permit number DWSP202009002, according to the National Guidelines on Animal Work in China. Female hACE2 transgenic mice were purchased from Shanghai Model Organisms. All work with infectious SARS-CoV-2 was performed with approval under biosafety level 3 (BSL3) and animal biosafety level 3 (ABSL3) conditions by the Institutional Biosafety Committee of the Institute of Medical Biology (IMB) in the Kunming National High-Level Biosafety Primate Research Center. To alleviate pain, all experiments were carried out in strict accordance with national guidelines for animal welfare. In accordance with the principle of animal ethics, humane euthanasia was performed.

### Crystallization and data collection.

Fab fragments were each mixed with SARS-CoV-2 RBD at a molar ratio of 1:1, incubated for 2 h at 4°C, and further purified by gel-filtration chromatography. The purified complex was concentrated to 11.55 mg/ml in buffer (20 mM Tris-HCl, pH 8.0, 150 mM NaCl [Sigma-Aldrich]) for crystallization. RBD-nCoV617 complex crystal seedings were grown in 0.2 M NH_4_Cl and 30% PEG4000 (Sigma-Aldrich). Screening trials were performed at 16°C. The hanging drop vapor diffusion method was used by mixing 1 μl of protein with 1 μl of reservoir solution and then adding 0.2 μl of 1/1,000 seedings. Crystals of RBD-Fab complexes were successfully obtained in 1/1,000 seedings in 152 mM NH_4_Cl and 22.8% PEG8000 (Sigma-Aldrich). Crystals were frozen in liquid nitrogen in reservoir solutions.

X-ray diffraction data were collected at the Shanghai Synchrotron Facility BL19U1 at a wavelength of 0.979 Å and a temperature of 100 K. The complex structure of NAb nCoV617 with S-RBD was determined by molecular replacement using the S-RBD structure (PDB: 6M0J) and monoclonal antibody C135 (PDB: 7K8Z) as the search model with the PHENIX software suite.

### Clash calculation between ACE2 and neutralizing antibodies.

The clash between neutralizing antibodies and SARS-CoV-2 RBD was calculated with Chimera. Once the RBD-ACE2 (PDB ID: 6M0J) was aligned to the RBD-NAb complexes, any residues that clashed were isolated and saved in a new coordinate file for volume calculation using the UCSF Chimera “measure volume” command.
