# Supplementary material for: Structural Basis of a Human Neutralizing Antibody Specific to the SARS-CoV-2 Spike Protein Receptor-Binding Domain
Source: Microbiol Spectr. 2021 Oct 13;9(2):e01352-21. doi: 10.1128/Spectrum.01352-21 (PMC8515945; doi:10.1128/Spectrum.01352-21)
Supplement: Supplemental file 1 — Supplemental material. Download SPECTRUM01352-21_Supp_1_seq9.pdf, PDF file, 0.5 MB [file spectrum01352-21_supp_1_seq9.pdf]

Supplementary Table

Table S1. Ig gene family of anti-tetanus toxin mAbs isoated.

Antibody ID	V_H							$V_{K/L}$				
	V_H ID	V	D	J	Mutation frequency	CDR3 length (No. of a.a)	Isotype	$V_{K/L}$ ID	V	J	Mutation frequency	CDR3 length (No. of a.a)
nCoV491	H16404	4-34	4-17	6	0.00%	18	IgA1	K12781	1-16	1	0.00%	9
nCoV497	H16419	3-30	1-26	4	2.08%	14	IgG1	K12793	3-20	4	0.35%	9
nCoV501	H16427	3-33	3-10	4	10.42%	11	IgG1	K12801	3-15	5	8.96%	9
nCoV503	H16431	3-23	3-16	4	3.12%	15	IgA1	K12805	4-1	5	3.37%	9
nCoV507	H16437	5-51	6-13	4	0.35%	13	IgG1	K12810	3-20	2	0.00%	9
nCoV511	H16451	3-48	2-2	4	0.69%	24	IgG1	K12818	1-39	1	1.43%	9
nCoV514	H16460	3-7	3-10	4	12.15%	12	IgG2	K12823	2-30	2	3.74%	9
nCoV526	H16497	4-30	1-14	3	14.78%	11	IgG2	K12848	1-39	1	8.96%	9
nCoV531	H16507	3-23	5-12	2	2.43%	13	IgG1	K12858	1-39	5	1.79%	10
nCoV543	H16536	1-2	3-9	3	0.69%	20	IgG1	K12880	1-39	1	0.72%	9
nCoV549	H16550	3-64	2-15	6	0.35%	12	IgG1	K12892	3-15	2	0.72%	10
nCoV556	H16565	1-69	3-22	5	0.00%	17	IgG1	K12906	3-20	4	1.42%	9
nCoV573	H16614	3-23	3-10	4	15.97%	14	IgA1	K12939	1-39	5	9.32%	9
nCoV581	H16638	3-7	2-21	4	11.11%	10	IgG2	K12955	3-20	4	10.99%	9
nCoV591	H16669	1-46	6-6	6	2.08%	20	IgA1	K12975	2-28	1	0.68%	9
nCoV597	H16686	3-30	1-26	4	1.04%	14	IgG1	K12987	3-20	4	0.35%	9
nCoV599	H16692	3-30	2-15	6	1.39%	14	IgG1	K12991	3-20	1	2.48%	9
nCoV600	H16694	1-69	2-2	6	0.35%	23	IgG1	K12994	3-20	1	0.35%	9
nCoV602	H16700	3-30	1-26	4	0.69%	13	IgG1	K12997	1-5	2	1.43%	5
nCoV604	H16710	4-34	2-2	3	0.00%	21	IgG1	K13001	1-33	2	0.00%	9
nCoV606	H16713	3-30	1-26	4	0.69%	14	IgG1	K13006	1-5	3	1.08%	8
nCoV608	H16380	4-30	3-10	6	4.47%	15	IgG1	L5462	1-44	1	1.75%	10
nCoV617	H16439	3-30	3-3	4	1.04%	16	IgG1	L5478	1-44	3	0.70%	10
nCoV626	H16487	1-2	1-1	4	6.25%	14	IgG4	L5494	2-8	3	2.78%	10
nCoV650	H16659	6-1	6-13	4	0.67%	16	IgG1	L5537	1-40	3	0.00%	11
nCoV653	H16690	1-2	1-26	6	0.35%	24	IgA1	L5541	2-14	2	0.00%	10
nCoV654	H16698	3-49	3-9	4	0.00%	22	IgG1	L5543	4-69	3	0.00%	9
nCoV657	H16708	3-30	5-18	4	0.69%	16	IgM	L5548	3-21	2	1.79%	9

Table S2. Data collection and refinement statistics.

	**SARS-CoV-2 S-RBD+nCoV617***
**Protein Data Bank ID**	7E3O
**Wavelength (Å)**	0.97853
**Resolution range**	40.67- 2.514 (2.603 - 2.514) **
**Space group**	$P\ 2_1\ 2_1\ 2_1$
**Unit cell**	
a, b, c (Å)	165.093 60.367 77.296
α, β, γ (°)	90, 90, 90
**Unique reflections**	25491 (2495) **
**Multiplicity**	4.9
**Completeness (%)**	94.20 (95.63) **
**Mean I/sigma (I)**	5.6
**Wilson B-factor**	53.99
**R-merge**[**]	0.118
**R-meas**[***]	0.132
**R-pim**[****]	0.056
**CC1/2**	0.897
**CC**	0.973
**Reflections used in refinement**	25465 (2495) **
**Reflections used for R-free**	1996 (196) **
**R-work**[#]	0.2202 (0.2810) **

**R-free**	0.2750 (0.3782) **
**CC (work)**	0.973
**Number of nonhydrogen atoms**	4751
Macromolecules	4697
Solvent	54
**Protein residues**	616
**RMS (bonds) (Å)**	0.011
**RMS (angles) (°)**	1.37
**Ramachandran favored (%)**	92.43
**Ramachandran allowed (%)**	6.74
**Ramachandran outliers (%)**	0.82
**Rotamer outliers (%)**	1.34
**Clashscore**	9.19
**Average B-factor**	53.91
Macromolecules	53.94
Solvent	51.89

*This dataset is collected with one crystal.

**Statistics for the highest-resolution shell are shown in parentheses.

Supplementary Figures

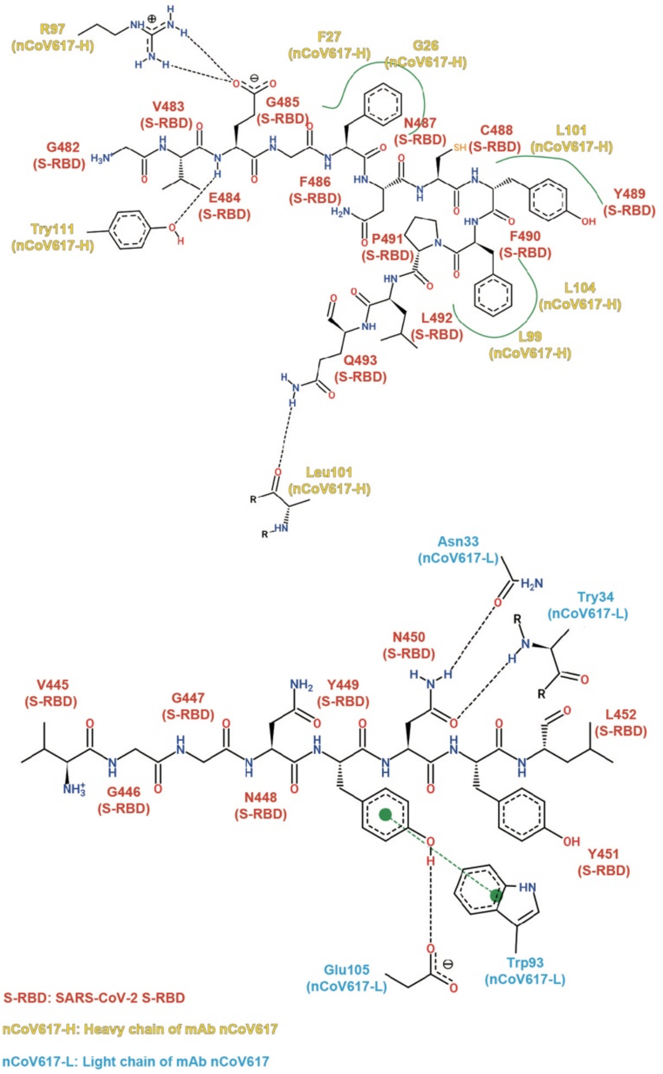

Figure. S1: Detail binding characterizations of residues in the complex. Residues in red color belong to SARS-CoV-2 S-RBD, in yellow color belong to heavy chain of mAb nCoV617, and in blue color belong to light chain of mAb nCoV617. Hydrogen bond is represented with black dash lines, while hydrophobic interaction is represented with green curves wrapped around the residues. The figure is generated by PoseView.

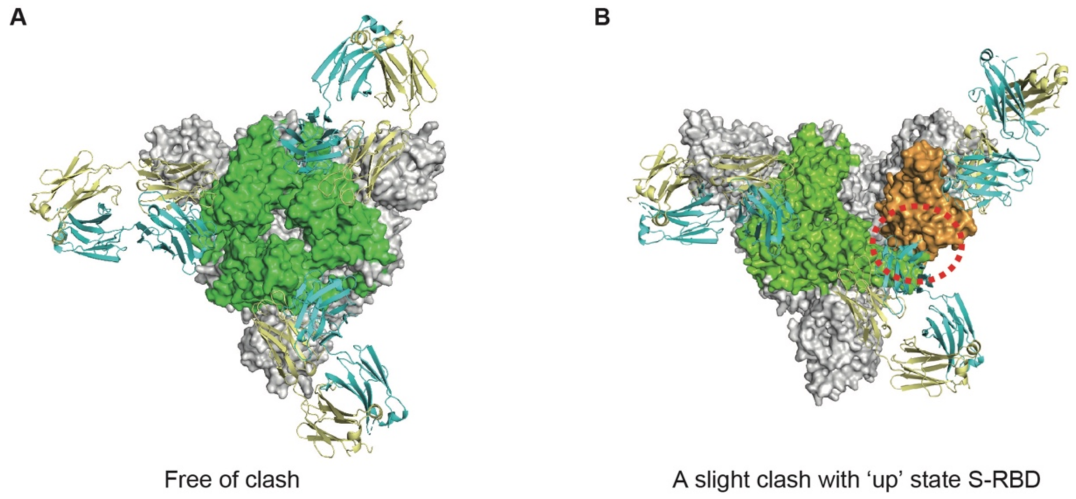

Figure. S2: Overall structure of nCoV617 Fab in complex with SARS-CoV-2 S protein (the 3-down(A), PDB ID: 6VXX; the 2-down-1-up(B), PDB ID: 7DF4). The green color indicate the down RBD in S protein, the orange color indicate the up RBD in S protein, the red dashed ellipse indicate the clash between nCoV617 Fab and S protein (PDB ID: 7DF4).
